# Alteration of Phenolic Composition in Lettuce (*Lactuca sativa* L.) by Reducing Nitrogen Supply Enhances its Anti-Proliferative Effects on Colorectal Cancer Cells

**DOI:** 10.3390/ijms20174205

**Published:** 2019-08-28

**Authors:** Weiwei Zhou, Xin Liang, Peibin Dai, Yao Chen, Yuxue Zhang, Miao Zhang, Lingli Lu, Chongwei Jin, Xianyong Lin

**Affiliations:** 1 MOE Key Laboratory of Environment Remediation and Ecological Health, College of Environmental and Resource Sciences, Zhejiang University, Hangzhou 310058, China; 2 Department of Applied Engineering, Zhejiang Economic and Trade Polytechnic, Hangzhou 310018, China; 3 Key Laboratory of Subtropical Soil Science and Plant Nutrition of Zhejiang Province, College of Environmental and Resource Sciences, Zhejiang University, Hangzhou 310058, China

**Keywords:** nitrogen, phenolic compounds, anti-proliferation, apoptosis, colorectal cancer, *Lactuca sativa* L.

## Abstract

Consumption of vegetables rich in phenolic compounds has become a useful method to reduce the risk of developing several types of cancer. This study investigated the potential relationship between the alteration of phenolic compounds in lettuce induced by reduced nitrogen supply and its anti-proliferative effects on Caco-2 colorectal cancer cells. Our results showed that phenolic extracts from lettuce grown under low nitrogen conditions (LP) exhibited better anti-proliferative effects against Caco-2 cells, in part, by interfering with the cell cycle and inducing apoptosis, compared with those from lettuce supplied with adequate nitrogen. High performance liquid chromatography (HPLC) analysis and correlation analysis indicated that the better anticancer activity of LP may be not only related to the increased phenolic content, but also associated with the increased percentage contribution of quercetin to total phenolics. Taken together, alteration of phenolic composition by reduced nitrogen supply can be an effectively strategy for the development of healthy vegetables as anticancer products.

## 1. Introduction

Colorectal cancer is the third most common form of cancer and the second-leading cause of cancer deaths worldwide [1]. The current treatment for colorectal cancer is usually surgical resection combined with radiation therapy and chemotherapy involving cytotoxic drugs. However, increases in the costs of health care and drug prices, along with the potential side effects of the aforementioned therapies, prompt research on alternative anticancer approaches. In recent years, epidemiological studies show that diets rich in vegetables are associated with a lower risk of chronic diseases, including cancer [2,3,4]. Phytochemicals such as phenolic compounds present in vegetables may contribute to this health-promoting effect. Phenolic compounds have been reported to decrease metastasis, induce apoptosis, and inhibit cell proliferation [5,6,7]. However, phenolic compounds are generally present in small quantities in vegetables under conventional cultivation conditions [8]. Therefore, from a nutritional standpoint, it is important to improve the accumulation of phenolic compounds in vegetables in the interests of human health.

Phenolic compounds are one of the most abundant groups of secondary metabolites in vegetables, generally synthesized through the phenylpropanoid pathway. This pathway is strongly influenced by many environmental factors, including temperature, light, and nutrient availability [9,10,11]. Nitrogen availability is an extremely important factor affecting the production of phenolic compounds in plants, with a reduction in nitrogen availability generally increasing their levels [12,13]. Our previous studies also found that proper nitrogen limitation could lead to increased accumulation of phenolic compounds in lettuce [14,15]. From an environmental standpoint, such a reduction promotes optimal efficiency in the use of nitrogen by plants, thereby limiting its loss in the environment. Phenolic compounds embrace a variety of structural classes such as phenolic acids, flavonoids, and anthocyanins, and possess a range of biological activities including antioxidant capacity, prevention of cardiovascular disease, and anticancer activity [1,16,17]. For example, an anticancer activity assay showed that caffeic acid exhibited a better anti-proliferative effect against cancer cells than chlorogenic acid [6]. However, to date, limited information exists regarding the relationship between the varied phenolic composition induced by the reduction of nitrogen availability and its anticancer activity, and the undermining mechanisms are also unclear.

Lettuce (*Lactuca sativa* L.) is the most common salad vegetable consumed worldwide. It is known to be a good source of health-promoting compounds, including phenolic compounds [3,18]. Importantly, lettuce is generally eaten fresh so that more ingredients remain intact. Consequently, increasing the phenolic content of lettuce is an important step toward accessing novel sources of natural phytochemicals. In the present study, we investigated the effects of reduced nitrogen supply on the production of phenolic compounds in lettuce, and further evaluated the anti-proliferative effects and underlying mechanisms of phenolic extracts from lettuce against Caco-2 cells. The results obtained may provide useful information about the feasibility of reduced nitrogen supply to modify phenolic composition in lettuce, thereby enhancing its anticancer activity.

## 2. Results

### 2.1. Reduced Nitrogen Supply Increases Phenolic Accumulation

**Figure 1:**
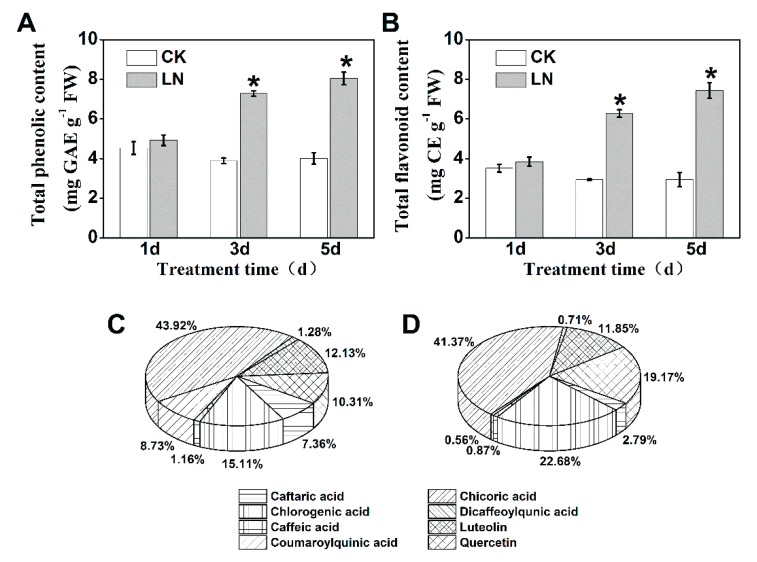
(**A**) Total phenolic content and (**B**) flavonoid content in lettuce cultivated under adequate (CK) or low nitrogen solution (LN). The percentage contribution of individual phenolic compounds to the total phenolics in lettuce extracts after 5 days of CK (**C**) or LN treatment (**D**). Data represent means ± SD (n = 6). * indicates significant difference at *P* < 0.05 between LN and CK.

A gradual increase of phenolic content was observed in leaves of lettuce plants that were subjected to prolonged low nitrogen treatment (LN; Figure 1A,B). After three days of LN treatment, the lettuce plants contained higher total phenolic and flavonoid contents, which increased by 100.50% and 153.06%, respectively, at the end of the experiment compared to the control (CK). HPLC analysis showed that the most abundant phenolic compound was chicoric acid, followed by chlorogenic acid, quercetin, and luteolin, and their concentrations increased by 156.7%, 309.0%, 406.7%, and 166.3%, respectively after five days of LN treatment (Figure S1). However, coumaroylquinic acid content decreased by 82.5% after LN treatment. The caftaric acid content was not affected by LN treatment. In addition, the percentage contribution of these individual phenolic compounds was also altered by the LN treatment (Figure 1C,D). The contribution of quercetin increased from 10.3% (CK) to 19.2% (LN), and that of chlorogenic acid increased from 15.1% to 22.7%, while other identified phenolic compounds showed decreased contribution to the total phenolic content.

**Figure 2:**
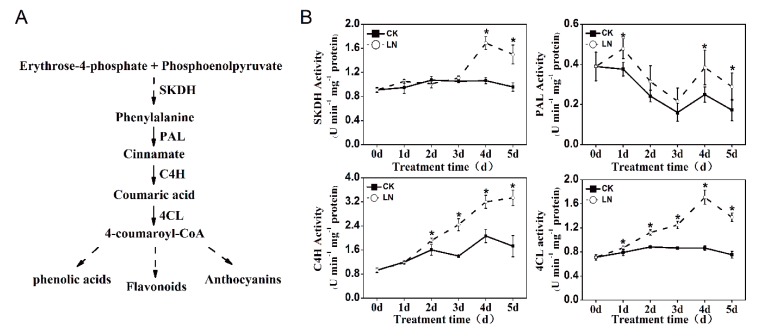
(**A**) Simplified scheme of the phenolic biosynthesis pathway and (**B**) the time course data of the activities of enzymes involved in phenolic biosynthesis in lettuce subjected to reduced nitrogen supply. The enzymes investigated are shikimate dehydrogenase (SKDH), phenylalanine ammonia-lyase (PAL), 4-coumarate coenzyme A ligase (4CL), and cinnamate 4-hydroxylase (C4L). Data represent are means ± SD (n = 6). * indicates significant difference at *P* < 0.05 between LN and CK.

As shown in Figure 2, the activities of enzymes involved in the synthesis of phenolic compounds were significantly affected by LN treatment. The shikimate dehydrogenase (SKDH) activity in LN-treated lettuce was higher on the 4th and 5th day by 58.66% and 56.45%, respectively, compared with the control. The activity of phenylalanine ammonia-lyase (PAL) in LN-treated lettuce was higher than that in control lettuce throughout the LN treatment, of which the significant increases were observed on the 1st, 4th, and 5th day. The cinnamate 4-hydroxylase (C4H) and 4-coumarate coenzyme A ligase (4CL) activities gradually increased with prolonged LN treatment, and their most strongly induced activity observed on the 5th and 4th day, respectively.

### 2.2. Lettuce Extracts Inhibit Caco-2 Cell Proliferation

**Figure 3:**
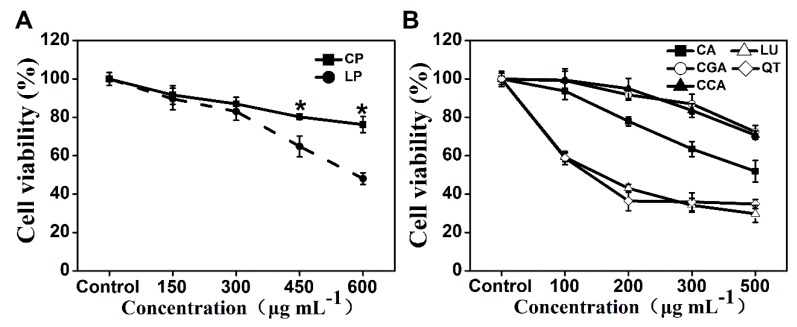
Effects of lettuce extracts (**A**) and pure individual phenolic compounds (**B**) on cell viability in Caco-2 cells. * indicates significant difference at *P* < 0.05 between LP and CP.

Treatment with lettuce extracts for 24 h induced a dose-dependent decrease in Caco-2 cell proliferation (Figure 3A). We observed that cell viability decreased to 64.84% and 48.08% in the presence of 450 and 600 µg mL^−1^ extracts from lettuce cultivated under low nitrogen solution (LP), respectively, compared to the control; however, identical treatments of extracts from lettuce cultivated under adequate nitrogen solution (CP) only decreased the cell viability to 80.20% and 76.23%, respectively. These results suggest that LP offers better anti-proliferative activities than CP. In order to evaluate the anticancer activities of the main phenolic compounds existing in lettuce extracts, Caco-2 cells were also treated for 24 h with five pure phenolic compounds (caffeic acid, CA; chlorogenic acid, CGA; chicoric acid, CCA; luteolin, LU; quercetin, QT) individually. Similar dose-dependent decrease in cell viability was also induced by treatment with the five phenolic compounds (Figure 3B). LU and QT exhibited similar effects on the viability of Caco-2 cells, and showed the highest anti-proliferative effects relative to other individual phenolic compounds. CGA and CCA also had similar effects on cell viability, but their anti-proliferative effects were lowest among the five phenolic compounds. The anti-proliferative effect of CA was lower than LU and QT, but higher than CGA and CCA. The correlation analysis also indicated that the phenolic compounds, except for coumaroylquinic acid, exhibited a negative correlation with cell viability, of which the largest correlation coefficient was found for luteolin and quercetin (Table 1).

### 2.3. Lettuce Extracts Induce Mitochondrial Membrane and DNA Impairments in Caco-2 Cells

**Figure 4:**
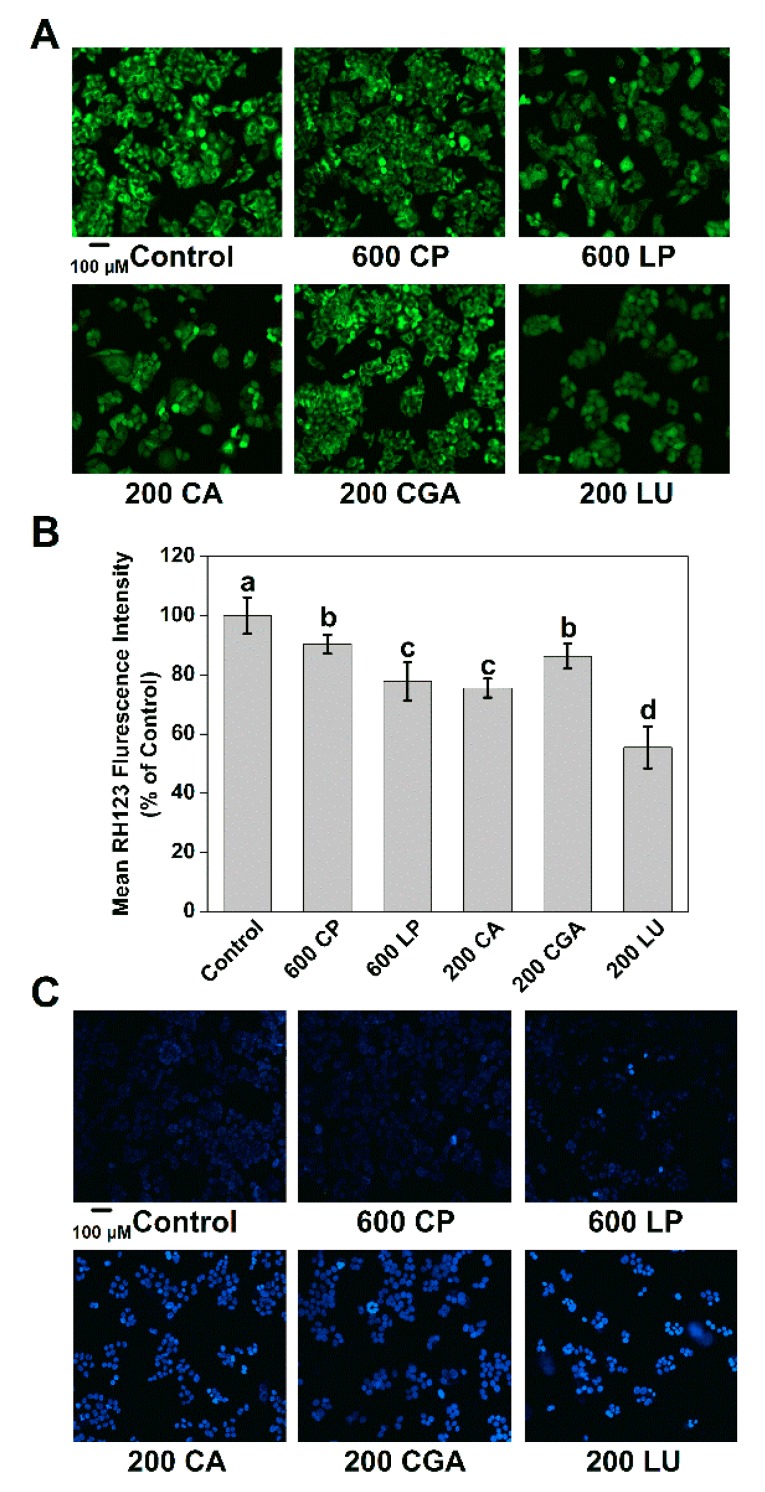
Effects of lettuce extracts and pure individual phenolic compounds on mitochondrial membrane potential and DNA damage in Caco-2 cells. (**A**) The mitochondrial membrane potential determined by fluorescence microscopy after incubation with RH 123. (**B**) The quantitative data of mean RH 123 fluorescence intensity. (**C**) Nuclear staining of Caco-2 cells with Hoechst 33258. Different letters indicate significant difference at *P* < 0.05.

As indicators of cell apoptosis, decreased mitochondrial membrane potential and DNA impairments were determined (Figure 4). Decreased intracellular RH 123 fluorescence intensity results from the efflux of the dye, which is in line with the loss of mitochondrial membrane potential. CP and LP (600 µg mL^−1^) treatment decreased the fluorescence intensity of RH 123 to 90.32% and 77.65%, respectively. CA, CGA, and LU (200 µg mL^−1^) treatment also decreased the fluorescence intensity to 75.78%, 86.30%, and 55.46%, respectively. Hoechst 33258 was used to assess nuclear damage (Figure 4C). After treatment with LP, CA, CGA, and LU for 24 h, the number of small bright blue dots representing chromatin condensation and fragmentation were increased compared with the control. Therefore, LP possesses a better biological composition for decreasing mitochondrial membrane potential and increasing DNA damage, compared with CP.

### 2.4. Lettuce Extracts Induce Caco-2 Cell Apoptosis and Cell Cycle Arrest

**Figure 5:**
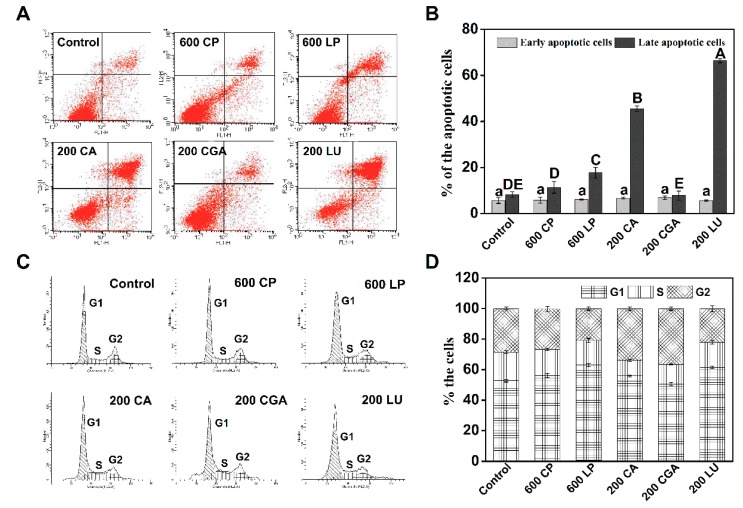
Effects of lettuce extracts and pure individual phenolic compounds on apoptosis and cell cycle in Caco-2 cells. (**A**) Representative images of flow cytometry apoptosis after 24 h treatments. (**B**) The percentage of early and late apoptotic cells. (**C**) Representative images of flow cytometry cell cycle after 24 h treatments. (**D**) Percentages in each cell cycle phase (G1, S, and G2). Different letters indicate significant difference at *P* < 0.05.

The population of apoptotic Caco-2 cells was significantly increased for both lettuce extracts and the pure phenolic compounds compared to the control (control: 13.91%; CP: 17.14%; LP: 23.81%; CA: 52.30%; CGA: 14.75%; LU: 72.06%) upon treatment for 24 h (Figure 5A,B). After CP, LP, CA, CGA, and LU treatment, more cells were in late apoptosis (11.33%, 17.72%, 45.56%, 7.83, and 66.40%, respectively) than early apoptosis (5.81%, 6.09%, 6.74%, 6.92%, and 5.66%, respectively). Next, cell cycle distribution was measured for the same treatments (Figure 5C,D). Compared to control, CP, LP, and LU treatment increased the percentage of Caco-2 cells in G1 phase by 6.13%, 19.16%, and 16.50%, respectively. CA and CGA treatments exhibited similar effects, simultaneously increasing the percentage of cells in G2 compared with the control. The results obtained indicate that lettuce extracts can inhibit the proliferation of Caco-2 cells by inducing cell apoptosis and cell cycle arrest, and the LP was more effective in the above process than CP.

### 2.5. Lettuce Extracts Change the Expression of Apoptosis Regulators in Caco-2 Cells

**Figure 6:**
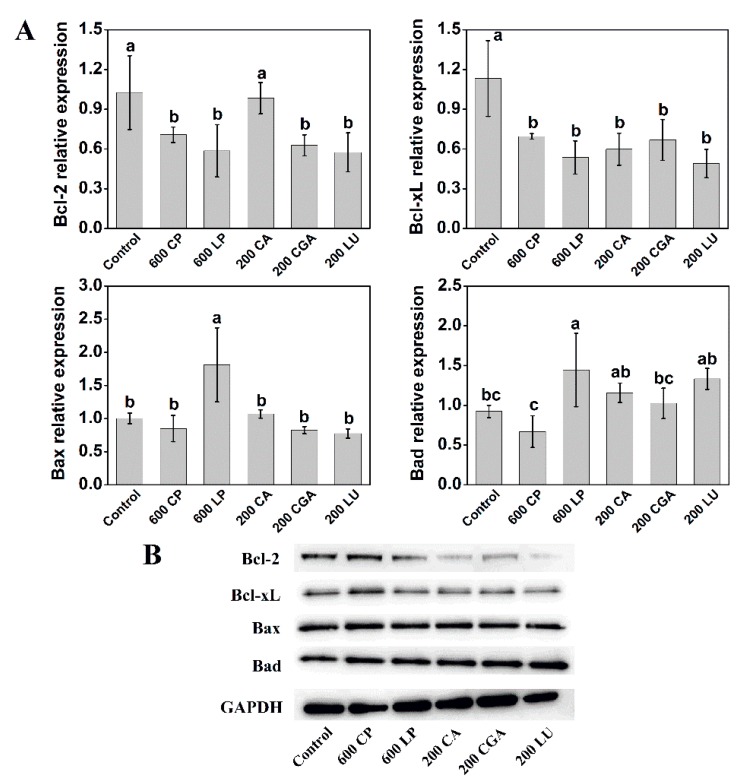
Effects of lettuce extracts and pure individual phenolic compounds on the expression of Bcl-2, Bcl-xL, Bax, and Bad as evaluated by RT-PCR analyses (**A**) and Western blotting (**B**), respectively, in Caco-2 cells. The relative transcript levels are given as a fold change relative to the control. Different letters indicate significant difference at *P* < 0.05.

The process of cell apoptosis involves the balanced transcription of anti-apoptotic and pro-apoptotic proteins. To test the mechanisms of cell apoptosis induced by phenolic extracts, the expression of apoptosis-related proteins in Caco-2 cells was further assessed. Treatment with LP (600 µg mL^−1^) decreased the transcriptional levels of Bcl-2 and Bcl-xL, two anti-apoptotic proteins, by 42.8% and 52.6%, respectively, compared with the control (Figure 6A). However, the CP (600 µg mL^−1^) treatment only resulted in a decrease in the transcriptional levels of Bcl-2 and Bcl-xL by 31.0% and 38.6%, respectively. On the contrary, Bax and Bad, two pro-apoptotic proteins, were up-regulated upon treatment with phenolic extracts, with higher transcriptional levels observed in LP than CP treatments. The CA, CGA, and LU (200 µg mL^−1^) treatment also decreased the transcriptional levels of Bcl-2 and Bcl-xL, and increased the transcriptional levels of Bad. The western blot analyses also revealed a similar trend in the protein expression of Bcl-2, Bcl-xL, and Bad, but no significant effects on Bax (Figure 6B).

## 3. Discussion

Cancer is a severe health problem that significantly undermines both life span and quality. Epidemiological studies suggest that a high intake of vegetables, especially those rich in phenolic compounds, results in a moderately reduced risk of cancer development [2,3,19]. Therefore, enhancement of the phenolic accumulation in vegetables would be beneficial for human health. Phenolic compounds are products of the phenylpropanoid pathway, which could be regulated by many environmental stresses [9,10,20]. Nitrogen is a critical player that regulates phenolic accumulation in plants, and generally low nitrogen promotes the accumulation of phenolic compounds [14,20,21]. In the present study, we found that reduced nitrogen supply not only increased the phenolic content in a time-dependent manner, but also altered the percentage contribution of these individual phenolic compounds identified in lettuce extracts (Figure 1). Quercetin and chlorogenic acid showed increased contribution to the total phenolics in lettuce extracts from LN treatment, while other individual phenolic compounds showed decreased contribution. This evolution of phenolic compounds was tightly correlated with the increased enzyme activity (SKDH, PAL, C4H, and 4CL) involved in the biosynthesis of phenolic compounds (Figure 2). SKDH, PAL, and 4CL activities reached their highest levels after four days of LN treatment, and then gradually decreased with prolonged treatment. An increase in PAL activity was also reported after four days of nitrogen deficiency in *Matricaria chamomilla* [22]. These results suggested that the effects of reduced nitrogen supply on phenolic accumulation were correlated, at least partially, with the increased activities of enzymes involved in the phenolic synthesis pathway.

The consumption of plant-derived phenolic compounds is associated with a decreased risk of developing many chronic degenerative diseases, including cancer. Decreased cancer cell proliferation has been observed after treatment with phenolic extracts from vegetables or fruits [2,4,17]. In present study, a dose-dependent decrease of Caco-2 cell proliferation was observed upon treatment with phenolic extracts from lettuce (Figure 3A). The inhibition of cancer cell proliferation can be characterized by morphological changes, such as a decline in mitochondrial membrane potential, nuclear fragmentation, and chromatin condensation [7,23,24]. We also found that phenolic extracts effectively decreased mitochondrial membrane potential and increased DNA damage in Caco-2 cells (Figure 4). Above morphological changes further confirmed the anti-proliferative effects of phenolic compounds extracted from lettuce on cancer cells. Interestingly, LP was more effective than CP in inhibiting Caco-2 cell proliferation. Consistent with our results, the superior performance of strawberry extracts with higher phenolic content in inhibiting cancer proliferation has been reported [25]. Our results revealed that LP had higher content of phenolic compounds than CP (Figure S1), and the phenolic compounds showed a high negative correlation with the cell proliferation (Table 1). Therefore, a possible explanation for the higher anticancer activity of LP might be attributed to the phenolic contents. In addition, we found that the five pure phenolic compounds identified in lettuce extracts showed varied anti-proliferative effects on Caco-2 cells, of which luteolin and quercetin exhibited greater anti-proliferative potency compared to caffeic acid, chlorogenic acid, and chicoric acid (Figure 3B). Our HPLC analysis further indicated that the quercetin and chlorogenic acid showed increased contribution to the total phenolics (Figure 1C,D). Based on above results, we suspected that the alteration of percentage contribution of phenolic compounds, especially quercetin, may also be partially responsible for the improved anticancer capacity of LP.

The anti-proliferative activity associated with the treatments of phenolic compounds could be mediated by the induction of cell cycle arrest and apoptosis. Anticancer compounds can arrest the cell cycle at the G1, S, or G2 phases to inhibit cell proliferation [4,5,6]. In the present study, lettuce extracts significantly increased the percentage of Caco-2 cells in G1 phase compared with the control, with more arrested cells in G1 phase observed for LP than CP (Figure 5C,D). A study looking at the effect of black lentil, sorghum, and red grape, high in anthocyanin, on human hepatocellular carcinoma cells also determined an increase in the percentage of cells in G1 phase [4]. Interestingly, pure luteolin treatment increased the percentage of cells in G1 phase, but caffeic acid and chlorogenic acid treatment increased the percentage of cells in G2 phase. The varied cell cycle arrest induced by individual phenolic compounds might together contribute to the anticancer performance of lettuce extracts. In addition to cell cycle arrest, we also found that lettuce extracts, caffeic acid, chlorogenic acid, and luteolin induced the apoptosis of Caco-2 cells, resulting in more cells in late apoptosis than early apoptosis (Figure 5A,B). In a study on purple corn, an increase in late apoptotic cells was seen when HT-29 cells were treated with corn extracts [26]. Apoptosis, a form of programmed cell death, can be regulated by the Bcl-2 family members. Responses to anticancer compounds might develop by the delicate balance between the relative levels of pro-apoptotic and anti-apoptotic members in the Bcl-2 family [1,7]. Our results showed that lettuce extracts and investigated pure phenolic compounds (caffeic acid, chlorogenic acid, and luteolin) decreased the expression of Bcl-2 and Bcl-xL, two anti-apoptotic proteins, and increased the transcriptional levels of Bax and Bad, two pro-apoptotic proteins (Figure 6). Importantly, LP was more effective than CP in regulating the expression of apoptosis-related proteins. Taken together, these results suggest that phenolic extracts derived from LN-treated lettuce were more effective in inhibiting Caco-2 cell proliferation by the induction of cell cycle arrest and apoptosis than those from control. One of the mechanisms through which many phytochemicals mediate cell growth and apoptosis is the scavenging or boosting of intracellular ROS [17,25,26]. However, the results of present study showed that there was no significant effect of lettuce extracts on the intracellular ROS levels in Caco-2 cell (Figure S2), implying that lettuce extracts-induced apoptosis might be not associated with ROS production.

In conclusion, this study showed that the modification of phenolic metabolism through reducing nitrogen supply not only effectively improved the accumulation of phenolic compounds of lettuce in a time-dependent manner, but also significantly altered the contribution of individual phenolic compounds to the total phenolics. Chlorogenic acids and quercetin showed increased percentage contribution in LP. These alterations of phenolic compounds in lettuce significantly affected the inhibitory potential of phenolic extracts on Caco-2 cells. The LP performed better in inhibiting Caco-2 cell proliferation, at least partially, by inducing cell cycle arrest and apoptosis than CP. Correlation analysis further demonstrated that the better performance of LP in inhibiting Caco-2 cells proliferation may be due to the increased phenolic compounds, especially quercetin. Taken together, the results from this study provide useful information for the development of vegetables with high bioactive phytochemicals through management of nitrogen availability.

## 4. Materials and Methods

### 4.1. Chemicals and Reagents

Folin-Ciocalteu’s phenol reagent, caftaric acid, caffeic acid, chlorogenic acid, chicoric acid, luteolin, quercetin, gallic acid, (+)-catechin, HPLC-grade methanol, formic acid, Hoechst 33258, 3-(4,5-dimethyl-2-thiazolyl)-2,5-diphenyl-2-H-tetrazolium bromide (MTT), and Rhodamine 123 were purchased from Sigma-Aldrich (St. Louis, MO, USA). Reactive Oxygen Species Assay Kit, Annexin V-FITC Apoptosis Detection Kit, Cell Lysis Buffer for Western and IP, BCA Protein Assay Kit, and Cell Cycle Analysis Kit were purchased from Beyotime Institute of Biotechnology, Ltd. (Shanghai, China). UNIQ-10 column TRIzol total RNA isolation kit was purchased from Sangon Biotech Co., Ltd. (Shanghai, China), and HiScript II Q RT SuperMix, ChamQ^TM^ SYBR Color qPCR Master Mix, and Chemiluminescence kit were purchased from Vazyme Biotech Co., Ltd. (Nanjing, China). Primary antibodies (anti-Bcl-2, anti-Bcl-xL, anti-Bax, anti-Bad, and anti-GAPDH) and secondary antibody (goat anti rabbit IgG (H+L)-HRP) were purchased from Diagbio Co., Ltd. (Hangzhou, China). All other chemicals used were of analytical grade or above.

### 4.2. Plant Material and Growth Conditions

Seeds of red pigmented lettuce (Ziluoma) were surface-sterilized using 80% (v/v) ethanol for 15 min, washed thrice, and rinsed in distilled water for about 12 h. The seeds were then transferred to a nylon net floating on 0.5 mM CaSO_4_ solution under a day/night temperature regime of 25/22 °C, a light intensity of 300 μmol m^−2^ s^−1^, and a relative humidity of 60%. Eight-day-old uniform seedlings were transplanted to a container with a nutrient solution consisting of 2 mM nitrate as the nitrogen source: 1.0 mM Ca(NO_3_)_2_, 2.0 mM K_2_SO_4_, 0.74 mM KH_2_PO_4_, and 1.0 mM MgSO_4_. Micronutrients were provided following the Hoagland solution formula. The solution was aerated continuously and renewed every four days. After 12 days, the uniform plants were transferred to 1.2-L plastic containers and divided into two groups for further treatments. One group was still cultured in 2 mM nitrate solution where the nitrogen supply is adequate for lettuce growth under present experimental conditions (data not shown), and another group was treated with low nitrogen solution. The low nitrogen solution (0.05 mM nitrate) contained only 0.025 mM Ca(NO_3_)_2_, 2.0 mM K_2_SO_4_, 0.74 mM KH_2_PO_4_, 1.0 mM MgSO_4_, and 0.975 mM CaCl_2_. The leaf samples were taken after 1, 3, and 5 days of reduced nitrogen treatment and immediately frozen by dipping in liquid nitrogen, then stored at −70 °C until further analysis.

### 4.3. Extraction of Phenolic Compounds

Phenolic compounds were extracted as described in previous studies with some modifications [27]. Lettuce leaves were homogenized in 80% (v/v) methanol, sonicated for 15 min at 4 °C, and centrifuged at 12 000 *g* for 10 min. The supernatant was then filtered through a 0.45 µm PTFE filter and evaporated to dryness under nitrogen gas. The residue was re-dissolved in chilled 10% dimethyl sulfoxide (DMSO) for further analysis.

### 4.4. Determination of Total Phenolic and Total Flavonoid Content

The concentration of total phenolic compounds was measured using the Folin-Ciocalteu assay [28,29]. Briefly, 0.3 mL of lettuce extract, 4.25 mL of H_2_O, and 0.25 mL of Folin-Ciocalteu reagent were mixed. After 3 min, 1.5 mL of 26.7% Na_2_CO_3_ and 2.7 mL of H_2_O were added, and the mixture was allowed to stand for 1 h. The absorbance was read at 760 nm using a spectrophotometer. Total phenolic content was expressed as gallic acid equivalent (GAE) in mg per g of fresh weight (FW).

The total flavonoid concentration was determined according to a method described by Jia et al [30]. In detail, 0.3 mL of extract was mixed with 2.2 mL of 30% ethanol and 0.15 mL of 5% NaNO_2_ for 5 min. Then 0.15 mL of 10% Al(NO_3_)_3_ was added to the mixture and incubated for an additional 6 min, followed by the addition of 1.2 mL of 30% ethanol and 1 mL of 5% NaOH. After incubation for 10 min, the absorbance was read at 510 nm. Total flavonoid content was expressed as (+)-catechin equivalent (CE) in mg per g of FW.

### 4.5. HPLC Analysis of Phenolic Compounds

High performance liquid chromatography (HPLC) was used to separate and quantify individual phenolic compounds in lettuce extracts. The extracts were separated on a C18 column (250 × 4.6 nm, 5 µm) at 35 °C using a water/methanol gradient. Solvent A consisted of 99% water and 1% formic acid, whereas solvent B contained 100% methanol. The following gradient was used: 0–15 min, 5% to 30% solvent B; 15–30 min, 30% to 50% solvent B; 30–35 min, 50% solvent B; 35–36 min, 50% to 60% solvent B; 36–40 min, 60% to 70% solvent B; 40–45 min, 70% to 90% solvent B; and 45–50 min, 90% solvent B. Elution was performed at a flow rate of 0.7 mL min^−1^ and the detector wavelength was set at 330 nm. The phenolic compounds identified were quantified based on comparison with external standards.

### 4.6. Analysis of Phenolic Synthesis-related Enzyme Activities

For the determination of shikimate dehydrogenase (SKDH) and cinnamate 4-hydroxylase (C4H) activities, fresh lettuce leaves (0.2 g) were homogenized in 100 mM potassium-phosphate buffer (pH 7.5) containing 0.5 mM dithiotreitol, 1 mM EDTA, 2 mM 2-mercaptoethanol, 1% polyvinyl poly-pyrrolidone, and 10% glycerol. For the determination of phenylalanine ammonia-lyase (PAL) and 4-coumarate coenzyme A ligase (4CL), fresh leaves (0.2 g) were homogenized in 100 mM Tris-HCl (pH 8.5) containing 0.5 mM dithiotreitol, 1 mM EDTA, 2 mM 2-mercaptoethanol, 1% polyvinyl poly-pyrrolidone, and 10% glycerol. Homogenates were centrifuged at 15 000 *g* for 15 min at 4 °C.

SKDH activity was assayed in a reaction mixture containing 1.45 mL of 2 mM shikimic acid and 0.5 mM NADP, as well as 0.1 mL of enzyme extract. Increasing absorbance due to the reduction of NADP was read over 1 min at 340 nm [31].

PAL activity was measured using a modified version of the method published by Tanaka et al [32]. The reaction mixture contained 1.4 mL of 120 µM L-phenylalanine and 50 µL of enzyme extract, which was incubated at 37 °C for 30 min; then the reaction was terminated by adding 6 M HCl. The same quantity of phenylalanine was added to the PAL assay control after termination. PAL activity was determined by the production of cinnamic acid. The absorbance of cinnamic acid was measured at 290 nm relative to the control.

C4H activity was assayed using the method described by Lamb and Rubery with slight modifications [33]. The reaction mixture contained 1.4 mL of reaction solution (containing 5 mM 2-mercaptoethanol, 5 µM trans-cinnamic acid, 0.5 µM NADPH, and 5 µM G-6-P-Na_2_) and 100 µL of enzyme extract, incubated at 37 °C for 30 min; then the reaction was terminated with 6 M HCl and readjusted to pH 11.0 with 6 M NaOH. The absorbance was measured at 290 nm.

The 4CL activity was determined using a spectrophotometric method with slight modifications [31]. The reaction mixture contained 0.95 mL reaction solution (containing 5 µM p-coumaric acid, 10 µM ATP, 5 mM 2-mercaptoethanol, 0.5 µM CoA-SH, 3 µM MgSO_4_) and 50 µL of enzyme extract, incubated at 40 °C for 10 min. The absorbance was then measured at 333 nm.

### 4.7. Cell Culture

Human Caco-2 cells were obtained from the Cell Bank of Type Culture Collection of Chinese Academy of Sciences. Caco-2 cells were cultured in DMEM containing 10% fetal bovine serum, 100 units mL^−1^ of penicillin, and 100 units mL^−1^ of streptomycin at 37 °C in a humidified cell incubator with 5% CO_2_.

### 4.8. Analysis of Cell Proliferation

The cell proliferation analysis was performed using the MTT method as reported previously [7]. The Caco-2 cells were seeded in a 96-well plate at a density of 5 × 10^3^ cells per well and then incubated for 12 h. After pretreatment with CP or LP (150, 300, 450, 600 µg mL^−1^) or pure phenolic compounds (CA, CGA, CCA, LU, and QT; 100, 200, 300, 500 µg mL^−1^) for 24 h, MTT at a concentration of 0.5 mg mL^−1^ was added to each well and incubated for another 4 h. Formazan precipitate was produced and dissolved in 150 µL of DMSO after discarding the supernatant. The absorbance was measured at 570 nm using a SpectraMax i3x microplate reader (Molecular Devices, San Jose, CA, USA).

### 4.9. Analysis of DNA Damage and Mitochondrial Membrane Potential

DNA damage was determined using Hoechst 33258 as described previously, with some modifications [34]. After treatment, the cells were incubated with 10 µM Hoechst 33258 at 37 °C for 30 min, washed with PBS, and immediately examined using a fluorescence microscope (Nikon Eclipse E600). The mitochondrial membrane potential (MMP) was monitored based on the fluorescence of RH 123 released from the mitochondria. The treated cells were incubated with 10 µg mL^−1^ of RH 123 at 37 °C for 30 min, washed with PBS, and immediately examined using a fluorescence microscope (Nikon Eclipse E600).

### 4.10. Analysis of Intracellular ROS Production

The level of cellular ROS formation was determined using the Reactive Oxygen Species Assay Kit. After treatment, the Caco-2 cells were collected and washed with PBS. The cells were incubated with 10 µM DCFH-DA at 37 °C for 30 min, and further washed three times with PBS. Fluorescence emitted at 538 nm with excitation at 485 nm was measured using a SpectraMax i3x microplate reader (Molecular Devices, Sunnyvale, USA).

### 4.11. Analysis of Apoptosis and Cell Cycle by Flow Cytometry

Cell apoptosis was determined using the Annexin V-FITC Apoptosis Detection Kit. After treatment, Caco-2 cells were collected, centrifuged for 5 min at 1500 rpm, and then washed twice with PBS. Cells were resuspended in binding buffer with Annexin V and propidium iodide (PI) for 15 min, and then analyzed by flow cytometry (BD FACSCalibur™ system, San Jose, USA).

For cell cycle measurement, the treated Caco-2 cells were collected and centrifuged for 5 min at 1500 rpm. The cells were resuspended in 70% ice-cold ethanol and stored overnight at 4 °C. The ethanol-suspended cells were centrifuged, washed with PBS, and then incubated at 37 °C for 30 min with PI and RNAase solution. The cells were analyzed by flow cytometry (BD FACSCalibur™ system). Data were plotted and analyzed using ModFit Software (Verity Software House, Inc., Topsham, ME, USA).

### 4.12. RNA Extraction and Gene Expression Analysis

Total RNA was extracted from Caco-2 cells using the UNIQ-10 column TRIzol total RNA isolation kit. The cDNA was synthesized using HiScript II Q RT SuperMix in a 10 µL reaction volume containing 500 ng purified RNA. Quantitative real-time PCR analysis was performed with ChamQ^TM^ SYBR Color qPCR Master Mix using a LightCycler 480 real-time PCR detection system (Roche, Rotkreuz, Switzerland). Specific primers for Bcl-2, Bcl-xL, Bad, and Bax were used as described in the previous studies [7,35]. Relative transcript levels were calculated against that of the internal GAPDH control (forward: 5ʹ-GTCTCCTCTGACTTCAACAGCG-3ʹ; reverse: 5ʹ-ACCACCCTGTTGCTGTAGCC-3ʹ) using the 2^-ΔΔCt^ method.

### 4.13. Western Blot Analysis

After treatment, the supernatant was removed and cells were washed twice with cold PBS. Then, the Caco-2 cells were lysed with Cell Lysis Buffer for Western and IP and the lysates were collected by scraping with a cold plastic cell scraper. The protein concentration of the supernatant was determined using the BCA Protein Assay Kit. SDS-PAGE electrophoresis was used to separate the proteins and then the proteins were transferred to PVDF membranes. After blocking with 5% milk, the proteins were detected by the proper primary and secondary antibodies before visualization using a Chemiluminescence kit. The primary antibodies were anti-Bcl-2, anti-Bcl-xL, anti-Bax, anti-Bad, and anti-GAPDH. The secondary antibody was goat anti rabbit IgG (H+L)-HRP.

### 4.14. Statistical Analysis

All data were analyzed using DPS software [36], with at least three experimental replicates. Significant differences were assessed using ANOVA and mean separation was determined using the least significant difference (LSD) test at *P* < 0.05. Fluorescence intensity was calculated using Image J (National Institutes of Health, Bethesda, MD, USA), an image analysis software. Fluorescence intensity percentages were calculated based on the percentage of the corresponding control group, for which the percentage was defined as 100%.

## Figures and Tables

**Table 1:** Correlation coefficients (r) between the cell viability and phenolic profiles. The phenolic profiles are total phenolic content (TP), total flavonoids content (TF) and the individual phenolic compounds in lettuce extracts.

Phenolic Compounds	Cell Viability
TP	−0.94 **
TF	−0.92 **
Caftaric acid	−0.10
Chlorogenic acid	−0.95 **
Caffeic acid	−0.96 **
Coumaroylquinic acid	0.99 **
Chicoric acid	−0.93 **
Dicaffeoylqunic acid	−0.74 *
Luteolin	−0.97 **
Quercetin	−0.97 **

Values designated by * are statistically significant at *P* < 0.05, and those denoted by ** are statistically significant at *P* < 0.01.

## Supplementary Materials

Supplementary materials can be found at https://www.mdpi.com/1422-0067/20/17/4205/s1.

## Abbreviations

CTA	caftaric acid
CA	caffeic acid
CGA	chlorogenic acid
CCA	chicoric acid
LU	luteolin
QT	quercetin
MTT	3-(4,5-dimethyl-2-thiazolyl)-2,5-diphenyl-2-H-tetrazolium bromide
SKDH	shikimate dehydrogenase
PAL	phenylalanine ammonia-lyase
C4H	cinnamate 4-hydroxylase
4CL	4-coumarate coenzyme A ligase
