# Supplementary material for: Alteration of Phenolic Composition in Lettuce (Lactuca sativa L.) by Reducing Nitrogen Supply Enhances its Anti-Proliferative Effects on Colorectal Cancer Cells
Source: Int J Mol Sci. 2019 Aug 28;20(17):4205. doi: 10.3390/ijms20174205 (PMC6747510; doi:10.3390/ijms20174205)
Supplement: Supplementary file 1 [file ijms-20-04205-s001.pdf]

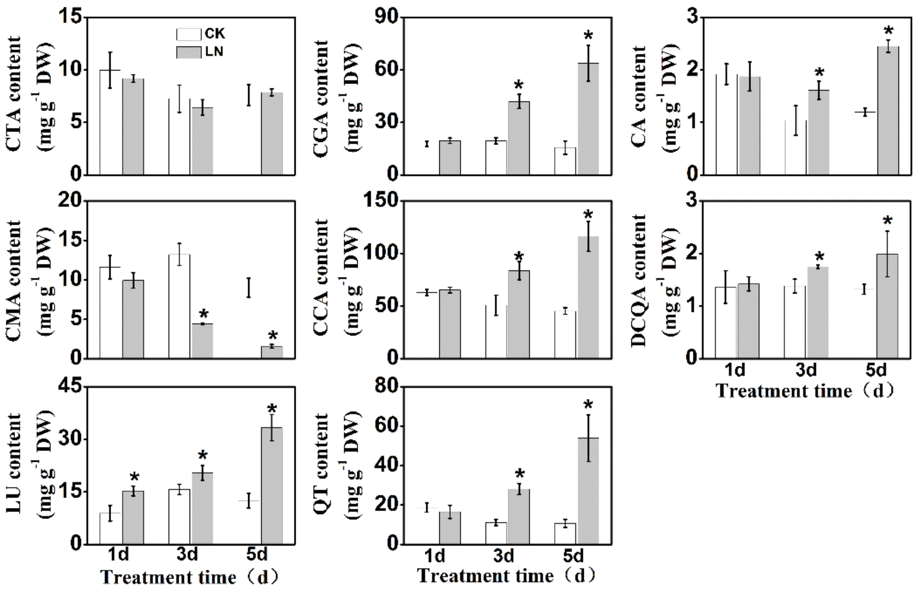

Figure S1. Contents of the individual phenolic compounds in lettuce extracts after treatment with reduced nitrogen supply. Caftaric acid (CTA), chlorogenic acid (CGA), caffeic acid (CA), coumaroylquinic acid (CMA), chicoric acid (CCA), dicaffeoylqunic acid (DCQA), luteolin (LU), quercetin (QT). * indicates significant difference at $P < 0.05$ between CK and LN.

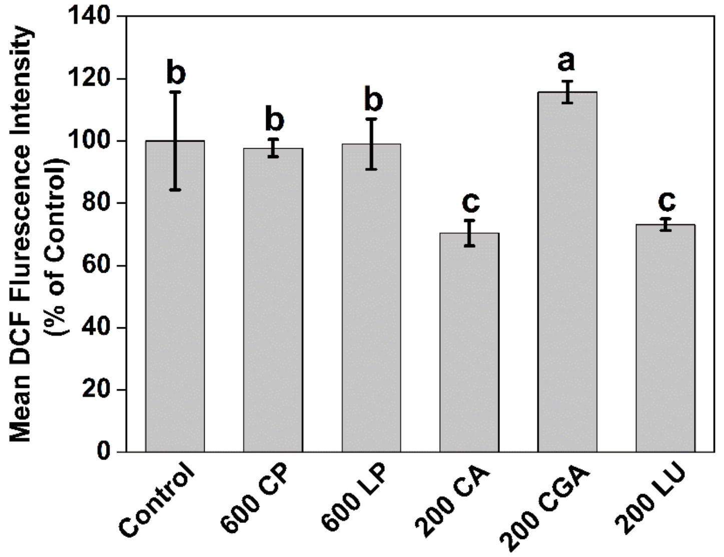

Figure S2. Effects of lettuce extracts and pure individual phenolic compounds on ROS production in Caco-2 cells. Different letters indicate significant difference at $P < 0.05$.
